# Water-Repellent Characteristics of Beech Wood Coated with Parylene-N

**DOI:** 10.3390/polym13132076

**Published:** 2021-06-24

**Authors:** Robert Köhler, Philipp Sauerbier, Mirco Weber, Roland-Christian Wander, Stephan Wieneke, Wolfgang Viöl

**Affiliations:** 1 Laboratory of Laser and Plasma Technologies, Faculty of Engineering and Health, University of Applied Sciences and Arts, Von-Ossietzky-Str. 99, 37085 Göttingen, Germany; mirco.weber@hawk.de (M.W.); roland-christian.wander@stud.hawk.de (R.-C.W.); stephan.wieneke@hawk.de (S.W.); wolfgang.vioel@hawk.de (W.V.); 2 Wood Biology and Wood Products, Faculty of Forest Sciences, University of Goettingen, Büsgenweg 4, 37077 Göttingen, Germany; psauerb@gwdg.de

**Keywords:** parylene-N, beech wood, CVD, wood protection, coating, water absorption

## Abstract

In recent years, awareness regarding sustainability and the responsible usage of natural resources has become more important in our modern society. As a result, wood as a building material experiences a renaissance. However, depending on the use case, protective measures may be necessary to increase wood’s durability and prolong its service life. The chemical vapor deposition (CVD) of parylene-N layers offers an interesting alternative to solvent-based and potentially environmentally harmful coating processes. The CVD process utilized in this study generated transparent, uniform barrier layers and can be applied on an extensive range of substrates without the involvement of any solvents. In this study, European beech wood samples (*Fagus sylvatica* L.) were coated with parylene-N using the CVD process, with paracyclophane as a precursor. The aim of the study was to analyze the water absorption of beech wood, in relation to the different layer thicknesses of parylene-N. Therefore, four different coating thicknesses from 0.5 to 40 μm were deposited, depending on the initial amount of precursor used. The deposited layers were analyzed by reflection interference spectroscopy and scanning electron microscopy, and their chemical structures and compositions were investigated by X-ray photoelectron spectroscopy and Fourier-transform infrared spectroscopy. Due to the chemical structure of parylene-N, the deposited layers led to a significantly increased water contact angle and reduced the water uptake by 25–34% compared to the uncoated reference samples. A linear correlation between layer thickness and water absorption was observed. The coating of wood with parylene-N provides a promising water barrier, even with thin layers.

## 1. Introduction

Wood, as a natural renewable raw material, is used in many fields of application. However, if wood is exposed to natural environmental influences, degradation of its components will occur. This is caused by, for example, UV radiation [1]. Additionally, the penetration of water causes the swelling and shrinkage of the wood [2], and the degradation products caused by the irradiation are washed out [3]. Furthermore, wood-decomposing fungi can penetrate the wood if the wood moisture content is sufficient [4]. Due to the water absorption and the decomposition processes, the mechanical properties are negatively affected and the appearance of the wood changes [5]. Wood can be protected against the described hazards through various approaches, such as impregnation or coating application.

In the individual processes, organic or inorganic materials are applied as barrier layers on the wood to prevent water uptake, block (UV) radiation and limit fungal growth [6,7].

An additional approach to protect substrates from water is to create parylene layers using chemical vapor deposition (CVD) [8]. There are four different types of parylene: N, C, D and F/AF4/HT. Parylene-N consists of an aromatic ring with attached methylene groups. In parylene-C and -D, one or two chlorine atoms are additionally bonded to the aromatic ring, respectively. Parylene-F/AF4/HT is a fluorinated variant of parylene-N [9]. All parylene forms possess good barrier properties due to the benzene structure, and are chemically inert to many substances, whereas the CVD process allows for homogeneous deposition and efficient crack sealing [10]. The deposited layers are in the amorphous and crystalline phase, which allows for good mechanical stability and low water permeability [11]. Even though parylene-C and parylene-F/AF4/HT possess better barrier properties against water [10,11], it is suggested to use parylene-N as a barrier to reduce water uptake into wood. Parylene-N is halogen-free, and therefore does not raise the same concerns for human health and the environment as halogenated hydrocarbons [12].

The aims of this study are to produce parylene-N layers of different thicknesses on European beech wood, and to investigate the chemical properties of the coating in order to determine their behavior as a water barrier. European beech wood was chosen because of its good availability in German forests, and with the aim of developing new fields for its usage [13].

## 2. Materials and Methods

### 2.1. Coating Process

European beech wood (*Fagus sylvatica* L.) samples (planed), with dimensions of 40 × 40 × 10 mm³ (radial × longitudinal × tangential), were used for the coating process. In preparation for the coating process, the samples were stored at 20 °C and 65% relative humidity (RH) until a constant mass was reached, which corresponds to a wood moisture content of approx. 12%. The samples’ densities were determined with a scale (Kern KB 360-3N, KERN & Sohn GmbH, Balingen, Germany) and a caliper, and were approx. 690 kg/m³.

To create different layer thicknesses (designation: C1, C2, C3 and C4), various amounts of paracyclophane (6 g, 20 g, 60 g and 100 g) were used inside the coating device, Lab-Coater 300 LV 35RR (Plasma Parylene Systems GmbH, Rosenheim, Germany), with a microwave generator as the plasma source. Due to the evaporation unit’s dimensions, it was impossible to coat the unit with 100 g of paracyclophane in one single process. Therefore, two similar coating processes with 50 g of paracyclophane each were performed. Before the coating processes started, 5 min of a low-pressure plasma glow discharge were applied to the samples with a power input of 300 W, gas flow of 300 sccm and ambient air as the process gas. After the plasma treatment, the coating chamber was evacuated to a pressure of 5.2 Pa and heated to a temperature between 45 and 50 °C. During the coating chamber evacuation, the evaporation unit was preheated to 60 °C and the pyrolysis unit to 690 °C. To start the coating process, the temperature of the evaporation unit was increased to 90 °C. After a significant decrease in pressure, the evaporation unit was heated up in the first step to 100 °C, and to 110 °C after the second pressure decrease. This temperature was applied for the duration it took to reach the starting pressure of 5.2 Pa. Afterwards, the coating chamber was vented, and the samples were removed from the coating device. Therefore, it was possible to coat all beech wood samples with equal parameters, with the exception of differences in coating time and the amount of paracyclophane used to obtain the various layer thicknesses.

For further investigations, the samples were stored after the coating process at 20 °C and 65% RH until a constant mass was achieved.

### 2.2. Sample Analysis

#### 2.2.1. Coating Thickness and Morphology

The layer thicknesses of the four different samples (*n* = 10) were determined using reflectometric interference spectroscopy (TranSpec Process Spectrometer, TranSpec, Aalen, Germany). The measurements were carried out in the spectral range between 600–980 nm with an integration time of 500 ms. To determine the layer thickness, the FTM ProVis software (TranSpec, Aalen, Germany) was used with the refractive index for parylene-N being set as 1.66.

The morphology of the uncoated beech wood and the parylene-coated samples were investigated using scanning electron microscopy (SEM, Microscope JEOL JSM-5600, at 15 kV, JEOL Ltd., Tokyo, Japan). In order to avoid charging effects, the samples were pre-sputtered with a gold layer approx. 15 nm thick.

#### 2.2.2. X-ray Photoelectron Spectroscopy

The chemical surface compositions were analyzed by high-resolution X-ray photoelectron spectroscopy (XPS) using a PHI VersaProbe II (Ulvac-phi, Inc., Chigasaki, Japan) with a monochromatic Al-Kα source, and photon energy of 1486.6 eV. The X-ray source used had a power of 100 W, whereby the sample surface was scanned with a beam size of 100 µm over an area of 1400 × 200 µm². High-resolution spectra of the coated and uncoated samples (*n* = 6) were recorded with a pass energy of 46.95 eV and a step size of 0.1 eV, with a constant electron take off angle of 45°. The spectrometer was calibrated to the copper and gold reference lines (932.62 eV and 83.96 eV), whereas the minimum detector resolution measured at the silver peak Ag (3d5/2) was 0.6 eV, with a pass energy of 23.5 eV. The measurements were obtained at room temperature and a base pressure of 2 × 10^−6^ Pa. To avoid charging effects, all measurements were carried out with charge compensation by utilizing a cold cathode electron flood source and low-energy argon ions. All spectra were shifted to the carbon peak (C1*s*) to 284.6 eV. The data analyses were performed using the software MultiPak (version 9.9.0.8, Ulvac-phi, Inc., Chigasaki, Japan).

#### 2.2.3. Fourier-Transform Infrared Spectroscopy

Fourier-transform infrared spectroscopy (FTIR) measurements for three samples of each variant were performed on a PerkinElmer Frontier (PerkinElmer LAS (Deutschland) GmbH, Rodgau Jügesheim, Germany). The measurements were performed, averaging 64 scans with a resolution of 4 cm^−1^, by utilizing a diamond ATR crystal in the range of 400–4000 cm^−1^. The obtained data were normalized to the maximum peak and baseline-corrected.

#### 2.2.4. Water Contact Angle

To determine the degree of water repellency, water contact angle measurements were carried out (MSA One-Click FSE Mobile Surface Analyzer, Krüss GmbH, Hamburg, Germany). Water, as a polar liquid which is relevant to wood’s durability, was applied with a drop volume of 0.5 µL, in 3 measurements onto 4 samples each (*n* = 12) for both the coated and uncoated samples. The corresponding software, ADVANCE (Krüss GmbH, Hamburg, Germany), was used to determine the contact angle with the Tangent 1 method.

#### 2.2.5. Water Uptake and Release Test of the Samples

The water absorption test was executed on the coated samples and the reference sample (*n* = 10) according to Meyer et al. [14]. The time-dependent water absorption after *t* = 5, 10, 20, 40, 60, 120, 240, 360, 1440 and 2880 min were determined with the following equation:

(1) $$W_{u}\left(t\right)=\frac{m_{t}-m_{0}}{m_{0}}\times 100,$$

with *W_u_* being the water uptake after time *t*, *m_0_* being the mass before the beginning of the immersion (*t* = 0), and m_t_ being the mass of the immersed samples at the time *t*.

The water release was calculated using the following equation:

(2) $$W_{r}\left(t\right)=\frac{m_{t}-m_{SUB}}{m_{SUB}}\times 100,$$

with *W_r_* being the water release after time *t*, *m_SUB_* being the mass after 2880 min of submersion in water, and *m_t_* being the mass of the dried samples (at 20 °C and 65% RH) at time *t*.

For the determination of the mass, a balance was used (Kern KB 360-3N, KERN & Sohn GmbH, Balingen, Germany).

#### 2.2.6. Statistical Analysis

To ensure a normal distribution (α = 0.05), a Kolmogorov–Smirnov normality test was used. The data were analyzed with Tukey’s honestly significant difference tests (α = 0.05) to identify statistical differences between the variants.

## 3. Results and Discussion

### 3.1. Coating Thickness and Morphology

**Figure 1:**
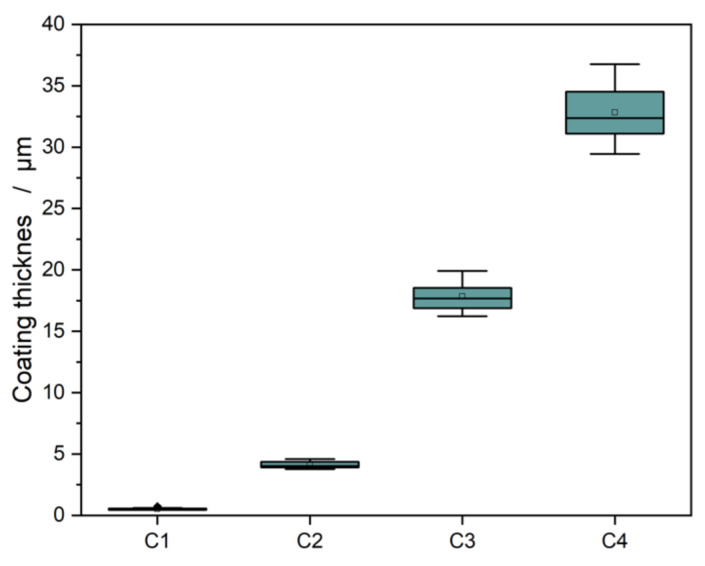
Coating thickness of the four different parylene layers.

The samples’ coating thicknesses were determined using reflectometric interference spectroscopy and are shown in Figure 1. The layer thicknesses ranged from 0.52 to 32.83 µm and were significantly different. With increasing amounts of the starting material (paracyclophane), the layer thickness also increased. With a layer thickness of 0.52 µm, the C1 layer was located at the pinhole boundary, where water reduction was still possible.

**Figure 2:**
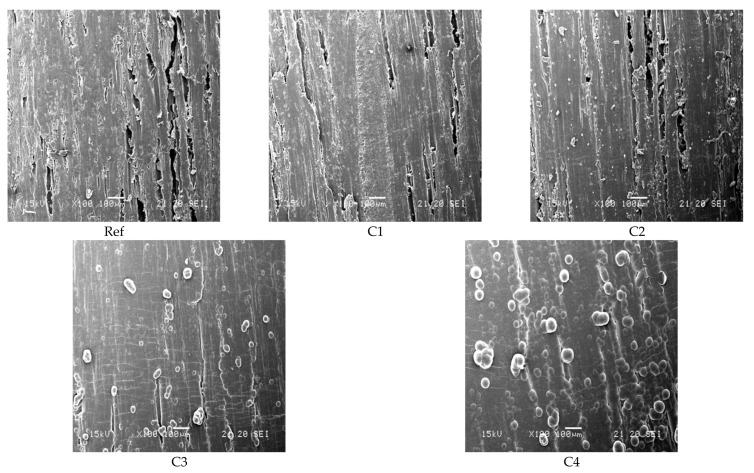
SEM images of the uncoated beech wood (**Ref**) and the parylene-coated (**C1**–**C4**) beech wood (magnification 100×).

To analyze the morphology of the reference samples and the different layers, SEM images were examined at a magnification of 100× (see Figure 2).

The uncoated beech reference (Ref) shows a typical wood surface with several vessels on the surface. The vessels can still be seen in the coated samples C1 and C2, and furthermore, the surface appears smoother, suggesting a good adaptation of the parylene to the surface. In C2, isolated agglomerates are visible which become more pronounced and more predominant with increasing layer thickness. In C3, only isolated vessels of the wood are visible, and a homogeneous layer covers the wood surface. A large amount of the precursor seems to fill the vessels. C4 also shows a closed layer with a higher percentage of agglomerates. The shape fidelity of the coating to the substrate is only evident in C1 and C2. The micro-cracks that appear across the grain course of the wood can be attributed to the sample preparation.

### 3.2. X-ray Photoelectron Spectroscopy (XPS)

**Figure 3:**
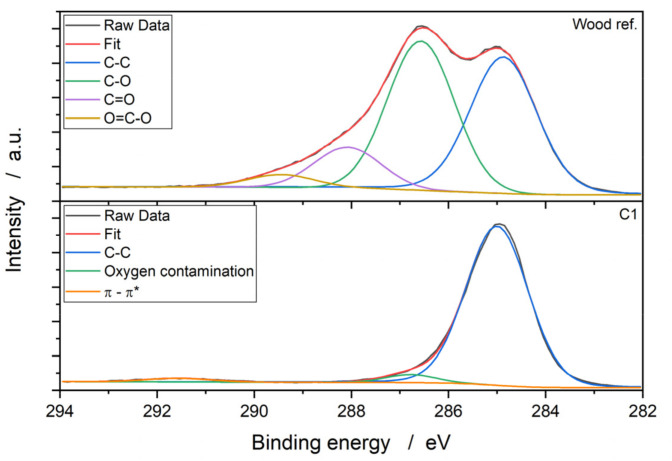
XPS carbon detail spectra of the uncoated wood reference and the parylene C1 coating (*n* = 6).

To determine the elemental composition and binding states, XPS measurements were recorded. The wood reference and the coatings essentially consisted of carbon and oxygen. In the case of the uncoated reference, nitrogen was also detected. Figure 3 shows the beech sample’s carbon detail spectrum and the C1 coating as a representative for the layers.

The C1*s* spectra of the beech wood shows four structures. The peak at around 285.0 eV could be attributed to the C-C and C=C bonds. The structures at 286.6 eV, 288.1 eV and 289.4 eV can be assigned to carbon-bound oxygen groups— specifically, to the C-O, carbonyl and carboxyl groups [15].

The resulting spectra of all the parylene coatings have the same nature, and consist of three peaks. The first peak at 285.0 eV could be assigned to chain and ring carbons, similar to the uncoated reference. The observed peaks at 286.8 eV and 291.6 eV show oxygen contamination [16] and the π–π* shake-up transition satellite of the aromatic ring system [17]. Specific decomposition of the carbon peak concerning the aromatic and the chain carbon peaks is difficult due to the closely spaced bond energies [16].

The chemical compositions of the wood reference and the coatings are shown in Table 1. The deposited layers have a similar composition to each other. Sodium was only additionally detected in C4, which can be attributed to impurities. Compared to the reference, the proportion of C-C and C=C increased by approx. 68 atom-%, while the oxygen content decreased to approx. 1.5 atom-%.

It can be assumed that the layers were deposited homogeneously on the wood surface. The significant reduction in oxygen concentration, along with the increase in carbon concentration and the peak shape of the C1*s* peak, are clear indicators of this assumption. The oxygen contaminations in the layers seem to be present only near the surface (limit of detection approx. 10 nm), which can be confirmed by the FTIR results below.

### 3.3. Fourier-Transform Infrared Spectroscopy (FTIR)

**Figure 4:**
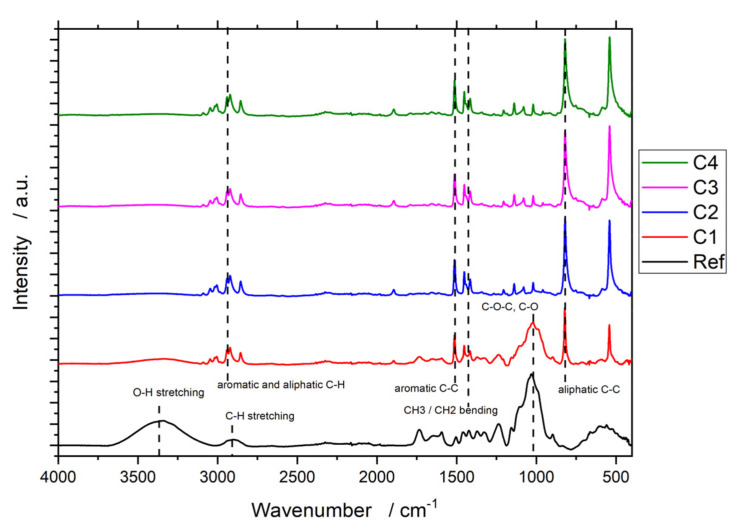
FTIR spectra of coated and uncoated beech wood.

FTIR results (Figure 4) of the untreated beech reveal an expected spectrum for lignocellulosic material. The spectrum has a broad O-H stretching peak around 3350 cm^−1^, a broad CH-stretching peak around 2900 cm^−1^, and C-O-C vibrational and C-O stretching peaks in the range of 1050 cm^−1^.

The resulting spectra for C2, C3 and C4 also show expected results for parylene-N. The peaks from 3050 to 2850 cm^−1^ are aromatic and aliphatic C-H stretching bands, the peak at 1513 cm^−1^ corresponds to the aromatic C-C stretching peak, and the double peak at 1420 cm^−1^ to the CH_3_/CH_2_ bending peaks. Finally, the peak at 820 cm^−1^ can be attributed to the aliphatic C−C stretching band.

While the reference spectrum is typical for beech wood and C2, C3, and C4 exhibit spectra that are expected for parylene-N measurement, the spectrum for C1 resembles a combination of both spectra.

Given the FTIR data, it is evident that the beech reference surface was covered with parylene-N. The peak intensity, especially of the O-H stretching peak around 3350 cm^−1^ and the C-O/C-O-C peaks around 1050 cm^−1^, decreased (and disappeared completely for C2, C3 and C4) when new peaks were formed. These new peaks are in accordance with the chemical composition of parylene-N, which is based on a benzene backbone connected by ethane in the para positions. According to Kahouli et al., the absence of carbonyl (theoretically expected to be around 1750 cm^−1^) and hydroxyl groups (theoretically expected to be around 3200 cm^−1^) in an FTIR spectrum is a sign of a high-quality deposited parylene-N film [18].

The C1 spectrum resembles a combination of the beech and the parylene-N spectra. However, this is not a sign of incomplete coverage of the surface of the beech wood. Due to the high depth of analysis for ATR measurement, the C1 coating thickness was not thick enough to prevent the measurement’s penetration into the underlying wood. The thickness of the C1 coating was determined to be 0.52 µm. However, the penetration depth for 1000 cm^−1^ is expected to be around 2 µm [19]. Therefore, the resemblance of the C1 spectrum to the beech reference and thicker parylene-N films agrees with what is expected theoretically for the FTIR measurements of thin films.

### 3.4. Water Contact Angle

**Figure 5:**
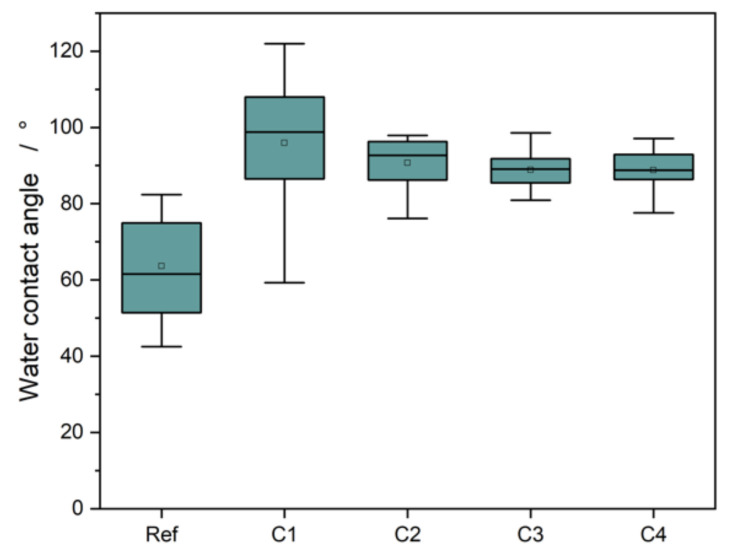
Water contact angles of the coated and uncoated beech wood. The coated wood surfaces showed an increase of the contact angle by 25–32°.

The measured water contact angles of the parylene layers and the beech reference can be found in Figure 5.

The initial water contact angle of the uncoated reference was 63.6 ± 13.2°. The coating process led to a significant increase in the water contact angle by 25–32°. One factor responsible for the increase in the contact angle was the coating’s chemical composition. As shown from the XPS measurements, the layers had a high fraction of aromatic and aliphatic carbon, which have non-polar properties. In this case, the increase in the non-polar fraction led to an increase in the contact angle [20].

### 3.5. Water Uptake and Release of the Samples

**Figure 6:**
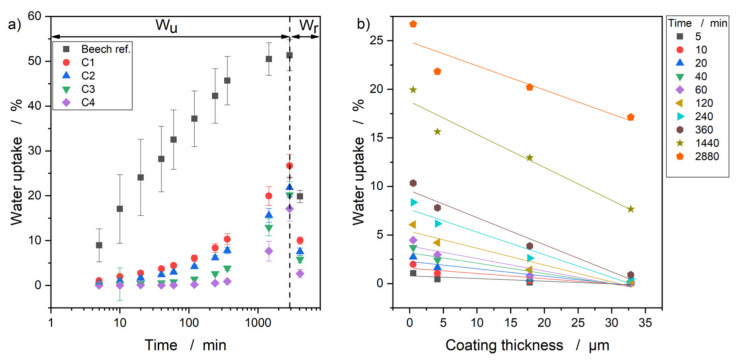
(**a**) Time course of water uptake (*W_u_*) and release (*W_r_*) of the coated and uncoated wood samples; (**b**) correlation of water uptake and coating thickness over the considered time period.

The time-dependent water uptake and release of the coated and uncoated wood samples are shown in Figure 6a. It should be mentioned that the water absorption test for the samples started at the equilibrium moisture content in the wood, which is 11–13%. The untreated wood sample showed a significant increase in water absorption in the first 6 h, and reached a plateau of approx. 50% after 24 h. All coated wood samples showed slowed water absorption over the observed period. After 48 h, a water absorption of 17–26% was observed, which corresponds to a reduction of 25–34% compared to the reference. After 48 h, the samples were dried at 20 °C and 65% RH. All samples showed a desorption behavior. The water release test was only performed for one measurement point to show that the coated substrates allowed for water release.

A comparison of the water uptake and the coating thickness is shown in Figure 6. A linear relationship can be observed between the layer thickness and the water absorption—with increasing thickness, the wood’s water absorption decreased.

The reduction in water uptake can be explained by capping/filling of the vessels with increasing precursor amounts, as observed in the SEM images. The C1 and C2 coatings also showed a reduction, which can be explained by the penetration of parylene into small crevices [21] (in this application wood vessels). The layers that filled the vessels showed better water reduction, which could be due to the insufficient penetration depth of the parylene and/or insufficient layer thickness. Another explanation could be that the penetration depth of parylene was sufficient but due to the shape fidelity, the pores still absorbed the water (capillary effect), which was reflected in a weight increase.

## 4. Conclusions

In this study, four different layer thicknesses of parylene-N were applied to beech wood in order to reduce the water uptake. The results of our investigations allow us to make the following conclusions:

- The deposition of parylene on wood was possible, whereby, depending on the treatment parameters, layer thicknesses of 0.5–32.8 µm were produced.
- All layers had the same chemical structure and consisted of an aromatic ring with attached methylene groups inside the monomeric unit. The XPS measurements revealed minor oxygen contamination on the coating surfaces, which can be attributed to the coating process.
- A true-to-shape coating could be produced with a layer thickness between 0.5 and 4.1 µm. With increasing parylene content, the filling of the vessels was observed.
- The applied coatings showed an increase in the water contact angle compared to the uncoated reference, leading to a water-repellent characteristic of the coated surface.
- For all layers, a reduction of water absorption was found, and a linear relationship between layer thickness and water absorption was detected. The water absorption decreased with increasing layer thickness.

Parylene-N is suitable for coating beech wood, whereby low layer thicknesses were sufficient to protect the wood from water. Different types of wood have different densities, structures and chemical compositions. Accordingly, further investigations should be carried out concerning the adhesion of the coatings, the water absorption and the cleavage of parylene in different wood species. In addition, further work should examine if the pretreatment of the wood (e.g., by sanding) is necessary to obtain better coating results. Furthermore, the possible use of parylene-C, -D, and -F/AF4/HT could be evaluated since these are expected to have even better water barrier properties.

## Figures and Tables

**Table 1:** Chemical composition in atom-% of the wood reference and the four different parylene layers (*n* = 6).

Sample	C-C, C=C	C-O	C=O	O-C=O	Oxygen Contamination	N1s	Na1s	O1s
Beech wood	27.17 ± 2.31	31.41 ± 1.03	9.01 ± 0.54	2.63 ± 0.31	–	0.34 ± 0.13	–	29.43 ± 1.47
C1	94.08 ± 1.65	–	–	–	4.04 ± 1.41	–	–	1.89 ± 0.27
C2	95.30 ± 0.61	–	–	–	3.15 ± 0.71	–	–	1.55 ± 0.17
C3	96.18 ± 0.70	–	–	–	2.52 ± 0.70	–	–	1.30 ± 0.32
C4	96.08 ± 0.32	–	–	–	2.23 ± 0.32	–	0.28 ± 0.18	1.41 ± 0.23

## Data Availability

The data presented in this study are available on request from the corresponding author.
